# Neurodevelopmental Risk in HIV-Exposed Uninfected Children: Call for Developmental Surveillance

**DOI:** 10.26502/jppch.74050227

**Published:** 2025-10-16

**Authors:** Aliyah Williams, Devendra K. Agrawal

**Affiliations:** Department of Translational Research, College of Osteopathic Medicine of the Pacific, Western University of Health Sciences, Pomona, California, 91766 USA

**Keywords:** Anti-retroviral therapy, Atazanavir, Developmental surveillance, Human Immunodeficiency virus (HIV), HIV-exposed uninfected children, Infection, Maternal-fetal environment, Maternal viremia, Mother-to-child transmission, Neurodevelopment, Psychomotor development

## Abstract

As global efforts to prevent mother-to-child transmission of HIV have expanded, the population of children born HIV-exposed, uninfected (HEU) has grown substantially. HEU children face distinctive biological and environmental exposures that may place them at risk for neurodevelopmental challenges. This narrative review synthesizes evidence from the past decade on the neurodevelopmental outcomes of HEU children, including cognitive, motor, and behavioral differences observed across multiple international cohorts. It explores contributing factors such as maternal immune alterations, residual viremia during pregnancy, antiretroviral drug exposure, and postnatal growth impairments. These exposures have been linked to measurable developmental delays in infancy and early childhood, with some effects persisting into school age. Despite these risks, developmental monitoring is not routinely included in the care of HEU children. This review underscores the need to integrate neurodevelopmental surveillance into pediatric HIV-exposed care, optimize anti-retroviral therapy regimens with consideration of fetal outcomes, and support caregiver- and community-based interventions that promote healthy development. Addressing the developmental needs of HEU children is critical to improving long-term outcomes and ensuring this growing population is not left behind.

## 1. Introduction

Due to prevention of mother-to-child transmission (PMTCT) programs, the incidence of pediatric human immunodeficiency virus (HIV) acquired through vertical transmission has decreased significantly. However, as a result, the number of children born HIV-exposed, uninfected (HEU) has grown substantially to approximately 15 million cases globally as of 2023 [1]. Despite their seronegative status, HEU children experience a distinct set of biological and environmental exposures that are often overlooked in pediatric care.

Current pediatric HIV care largely focuses on HIV-exposed, infected (HEI) children, while HEU children typically fall outside the scope of neurodevelopmental monitoring and intervention. Yet, over the last decade, studies from diverse global settings have documented consistent differences in the neurodevelopment of HEU children when compared to their HIV-unexposed, uninfected (HUU) peers. Whether due to in utero exposure to maternal immune activation, antiretroviral therapy (ART), or both, HEU children grow within a context of neurodevelopmental vulnerability [2,3], suggesting that they may benefit from the same type of early, proactive behavioral and educational support offered to other high-risk pediatric populations.

This article aims to synthesize the current evidence on the neurodevelopmental impact of perinatal HIV exposure in uninfected children. In doing so, it aims to highlight the pressing need for routine developmental surveillance and integration of behavioral interventions into the standard care of HEU children, especially in regions most affected by the HIV epidemic.

## 2. Immune Dysregulation in the Maternal-Fetal Environment

Prenatal exposures unique to HEU children may place them at increased risk for subtle but meaningful neurodevelopmental differences. One biologically plausible contributor is immune dysregulation in the maternal-fetal environment, driven by maternal HIV infection. Chronic immune activation and altered cytokine signaling during gestation can interfere with critical neurodevelopmental processes, such as neuronal proliferation, migration, and synaptic pruning. Supporting this, a longitudinal study from South Africa found that HIV-infected mothers and their HEU infants exhibited persistently lower levels of IFN-γ, IL-1β, IL-2, and IL-4 during the first two years of life, alterations that were significantly associated with poorer gross motor development at 24 months [4]. These findings suggest that HIV-related immune shifts in utero and early infancy may contribute to developmental vulnerability, even in the absence of direct infection.

## 3. Maternal Viremia and Neurodevelopment

Even with anti-retroviral therapy (ART), some HIV-infected mothers may have residual viremia during pregnancy, which can expose the developing fetus to inflammatory cytokines and viral byproducts. This pro-inflammatory intrauterine environment may interfere with early brain development, including processes like myelination and synapse formation. Supporting this, a prospective cohort study found that higher maternal HIV RNA levels during pregnancy were associated with significantly lower motor and expressive language scores in HEU children at 24 months, even after adjusting for socioeconomic and psychosocial confounders [5]. These findings highlight that despite effective viral suppression with ART; low-level maternal viremia may still contribute to neurodevelopmental vulnerability in HEU children.

## 4. Anti-retroviral Therapy Exposure and Timing

Beyond its critical role in preventing vertical transmission, ART may also impact fetal development through direct and indirect biological mechanisms. Several ART regimens can cross the placenta and interfere with neurodevelopment, metabolism, growth, and bone health [6]. Specifically, exposure to tenofovir disoproxil fumarate (TDF) has been linked to mitochondrial dysfunction and subsequent neurocognitive delays.

The type of ART and timing of initiation also appears to influence developmental outcomes. For example, in utero exposure to atazanavir (ATV) was associated with a 70% increase in the odds of neurodevelopmental concerns when ART was initiated post-conception (cOR: 1.70, 95% CI: 0.82–3.54), with the risk rising substantially when started in the first trimester (cOR: 5.81, 95% CI: 2.41–14.0) [3].

Additionally, among HEU children, maternal ART initiation during the third trimester or later was linked to significantly lower Psychomotor Development Index (PDI) scores compared to initiation before pregnancy or in the first two trimesters (P = 0.027). These findings highlight the need for routine developmental monitoring in HEU children and careful selection and timing of ART during pregnancy to support optimal neurodevelopment.

## 5. Evidence of Neurodevelopmental Outcomes in HEU Children

Emerging literature from various international cohorts has documented measurable neurodevelopmental disparities between HEU and HUU children. These disparities span cognitive, motor, and behavioral domains, often manifesting within the first two years of life and sometimes persisting into childhood.

Several cohort studies report that HEU children score lower across multiple developmental domains, including language, fine, and gross motor skills, and social-emotional functioning. For example, a cohort study in Botswana found that HEU children scored significantly lower on receptive and expressive language measures of the Bayley Scales of Infant Development, Third Edition (Bayley-III), compared to HUU children, even after adjusting for maternal education and household income [7]. Similarly, in a South African cohort, HEU children demonstrated lower total and cognitive scores on the Bayley-III compared to HUU children, despite being medically stable and receiving routine postnatal care [8].

**Figure 1:**
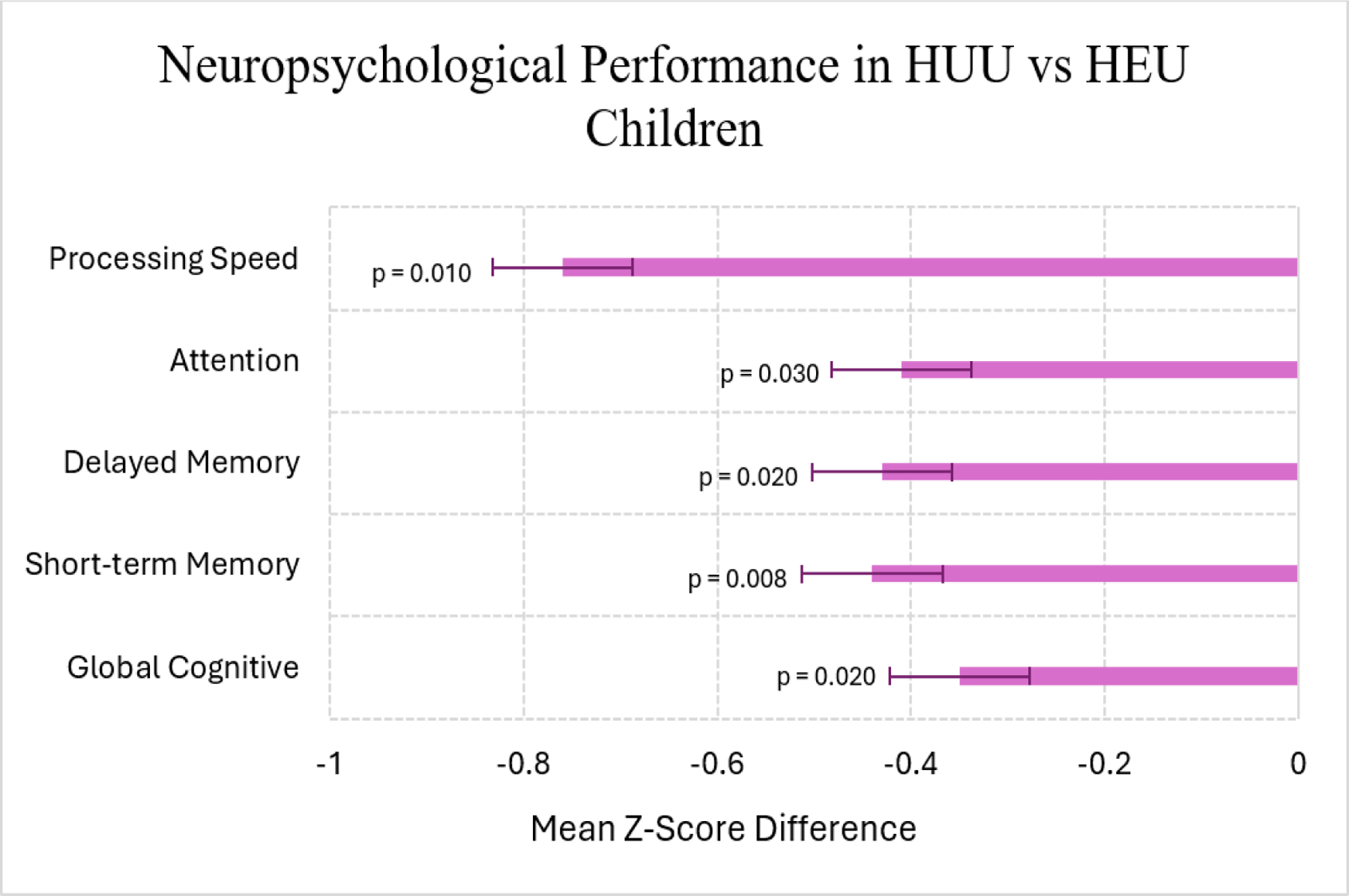
Comparison of adjusted mean z-scores for neuropsychological domains between HEU and HUU children based on the data from Benki-Nugent et al. [9]. HEU children demonstrated significantly lower scores across several neuropsychological domains (all p < 0.05). Error bars represent 95% confidence intervals.

Among older children, a Kenyan study of students (5–12 years) showed that HEU children performed worse than their HUU counterparts across several neuropsychological domains as illustrated in Figure 1. Importantly, these differences remained statistically significant even after controlling for age, sex, caregiver education, and household socioeconomic status [9].

Expanding on these findings, research highlights specific vulnerabilities within HEU subpopulations. For instance, a large U.S.-based study of 678 HEU infants found that HEU children born to women with perinatally acquired HIV (PHIV) exhibited lower language (mean score 91.9 vs. 94.8, p = 0.05) and motor (93.7 vs. 96.8, p = 0.03) composite scores on the Bayley-III compared to infants born to women with non-perinatally acquired HIV (NPHIV) [10–12]. These results emphasize that certain HEU subgroups may be at heightened risk for developmental delays.

Collectively, these findings suggest that biological exposures unique to HEU children contribute to early developmental challenges, reinforcing the imperative need for proactive and ongoing neurodevelopmental monitoring to identify and address potential cognitive delays promptly.

## 6. Physical Growth Impairments and Neurodevelopment

Physical growth impairments are frequently observed among HEU children and are closely linked to neurodevelopmental outcomes. In a Ugandan cohort, 38% of HEU infants experienced failure to thrive by 18 months, and lower anthropometric measurements were significantly associated with poorer developmental scores on standardized tests including the Malawi Development Assessment Tool (MDAT) and the Color Object Association Test [13–15]. Further emphasizing this connection, a second Ugandan study using longitudinal data found that HEU infants had significantly greater odds of falling into the lowest length-for-age (LAZ) and fat mass growth trajectories (FMGT) compared to HUU infants (LAZ OR=3.80 [1.22–11.82] and 8.72 [1.80–42.09]; FMGT OR=3.85 [1.39–10.59]) [16–18]. Together, these two Ugandan studies provide converging evidence from both developmental assessments and body composition analyses, emphasizing the clinical significance of postnatal growth monitoring in this regional population.

Furthermore, a Zimbabwean study identified significantly lower insulin-like growth factor 1 (IGF-1) levels in HEU infants at six weeks compared to their HUU counterparts, with IGF-1 levels correlating with subsequent linear and ponderal growth delays [16]. Given the critical role of IGF-1 in early brain development [17], these discrepancies may help explain the observed relationship between physical growth impairment and neurodevelopmental delays in HEU children.

Importantly, longitudinal data further suggests that growth-related disparities may persist into later childhood. In a Zambian study of school-aged children (6–12 years), HEU children exhibited significantly lower mid-upper arm and hip circumferences, and reduced body fat percentages compared to their HUU peers. Even after adjusting for demographic factors, HEU children scored significantly lower in mathematics, reinforcing a potential link between early-life exposures, chronic growth restriction, and cognitive performance [19]. Collectively, these findings highlight that poor postnatal growth in HEU children may serve as an early clinical indicator of broader neurodevelopmental vulnerability.

## 7. Clinical Implications and Recommendations

While HEU children are uninfected, they are certainly not unaffected. The current findings emphasize the need to recognize HEU children as a distinct pediatric population with specific developmental vulnerabilities. Even in settings with optimized medical care, studies consistently show delays in language, motor, and cognitive development among HEU children [15,20,21].

**First**, neurodevelopmental surveillance should be routinely integrated into pediatric HIV-exposed care models, regardless of infection status. Routine developmental screening during infancy and early childhood can enable earlier identification of at-risk HEU children and timely referral for interventional services. Validated tools such as the MDAT are feasible for use in low-resource settings and could be incorporated into standard well-child visits for HIV-exposed populations [15].

**Second**, growth monitoring should be treated as more than a marker of nutritional status. Evidence persistently shows that postnatal growth faltering is closely linked to developmental challenges [15,18,19]. Tracking simple anthropometric measures like LAZ and mid-upper arm circumference can serve as practical, cost-effective early warning signs that warrant further developmental evaluation and support.

**Third**, maternal care during pregnancy remains critical. Findings indicate that maternal viremia, timing of ART initiation, and specific ART regimens can impact fetal brain development [22–24]. Optimizing ART protocols not only for viral suppression but also to minimize fetal neurotoxicity should be a continued research and clinical priority.

**Lastly**, community-based and caregiver-focused interventions should be leveraged to support early childhood development. Parenting interventions and early stimulation programs have demonstrated efficacy in improving cognitive outcomes in children facing adversity and may be particularly beneficial for HEU children experiencing intersecting biological and social risks [25–27].

## 8. Discussion

While recent narrative reviews have broadly summarized the overall health and developmental profiles of HIV-exposed uninfected (HEU) children [14], the present review focuses specifically on neurodevelopment. It synthesizes evidence from the past decade on cognitive, motor, and behavioral outcomes, with particular attention to the biological mechanisms—such as impaired growth trajectories, inflammation, and altered maternal immunity—that may underlie these disparities.

This focused lens helps clarify that the neurodevelopmental challenges faced by HEU children are not incidental but stem from a complex interplay of prenatal and postnatal factors. By highlighting these mechanisms, this review reinforces the idea that HEU children should not be considered developmentally equal to their unexposed peers, even when they are medically stable and seronegative.

Despite advancements in preventing perinatal HIV transmission, the developmental outcomes of HEU children remain a neglected dimension of pediatric HIV care. Current pediatric HIV guidelines largely prioritize infection status, with limited integration of developmental monitoring in routine follow-up. As the global HEU population continues to grow, there is an urgent need to reframe care models to account for neurodevelopmental risk and resilience.

In addition, while this review focuses on biological contributors, environmental and structural determinants—such as caregiver mental health, poverty, and stigma—likely interact with biological risk to shape developmental outcomes. Future studies should adopt longitudinal and interdisciplinary approaches that consider both medical and social dimensions of HEU development.

## 9. Conclusion

HEU children face unique neurodevelopmental challenges that warrant focused clinical and research attention. As their numbers continue to grow globally, particularly in resource-limited settings, increased efforts from clinicians, researchers, and policymakers are essential. Longitudinal, multidisciplinary research must continue to elucidate underlying mechanisms and refine intervention strategies. Meanwhile, integrating neurodevelopmental care into existing health systems and leveraging community-based support can help ensure that HEU children achieve their full developmental potential and thrive despite the challenges posed by HIV exposure.

## 10. Key Points

- Globally, ~15 million children are HIV-exposed, uninfected (HEU), yet their neurodevelopmental needs are often overlooked in pediatric HIV care
- HEU children face unique prenatal exposures including maternal immune dysregulation, residual maternal viremia, and in utero ART exposure, which may disrupt brain development
- Cohort studies consistently report lower cognitive, motor, and language scores in HEU children compared to HIV-unexposed peers
- Certain HEU subgroups remain at heightened risk for developmental delays
- Postnatal growth impairment is common in HEU children and is strongly correlated with poorer neurodevelopmental outcomes
- Maternal viremia, even at low levels under ART, has been linked to lower motor and language scores in HEU infants
- Timing and type of ART exposure are associated with greater developmental concerns in HEU children
- Neurodevelopmental surveillance is not routinely integrated into HEU pediatric care, despite evidence of measurable risk
- Simple, low-cost measures such as growth monitoring and validated developmental screening tools (i.e., MDAT) could provide early detection of at-risk HEU children
- Community- and caregiver-based interventions show evidence for mitigating developmental vulnerabilities and should be integrated into care models
